# An effective approach for gap-filling continental scale remotely sensed time-series

**DOI:** 10.1016/j.isprsjprs.2014.10.001

**Published:** 2014-12

**Authors:** Daniel J. Weiss, Peter M. Atkinson, Samir Bhatt, Bonnie Mappin, Simon I. Hay, Peter W. Gething

**Affiliations:** a Spatial Ecology and Epidemiology Group, Tinbergen Building, Department of Zoology, University of Oxford, Oxford, UK; b Geography and Environment, University of Southampton, University Road, Southampton SO17 1BJ, UK; c Fogarty International Center, National Institutes of Health, Bethesda, MD, USA

**Keywords:** Gap-filling, MODIS, EVI, LST, Africa

## Abstract

The archives of imagery and modeled data products derived from remote sensing programs with high temporal resolution provide powerful resources for characterizing inter- and intra-annual environmental dynamics. The impressive depth of available time-series from such missions (e.g., MODIS and AVHRR) affords new opportunities for improving data usability by leveraging spatial and temporal information inherent to longitudinal geospatial datasets. In this research we develop an approach for filling gaps in imagery time-series that result primarily from cloud cover, which is particularly problematic in forested equatorial regions. Our approach consists of two, complementary gap-filling algorithms and a variety of run-time options that allow users to balance competing demands of model accuracy and processing time. We applied the gap-filling methodology to MODIS Enhanced Vegetation Index (EVI) and daytime and nighttime Land Surface Temperature (LST) datasets for the African continent for 2000–2012, with a 1 km spatial resolution, and an 8-day temporal resolution. We validated the method by introducing and filling artificial gaps, and then comparing the original data with model predictions. Our approach achieved *R*^2^ values above 0.87 even for pixels within 500 km wide introduced gaps. Furthermore, the structure of our approach allows estimation of the error associated with each gap-filled pixel based on the distance to the non-gap pixels used to model its fill value, thus providing a mechanism for including uncertainty associated with the gap-filling process in downstream applications of the resulting datasets.

## 1 Introduction

Past and current data collection efforts have produced numerous remotely sensed imagery time-series, often exceeding a decade in length, with tremendous utility (both realized and potential) for a wide range of research applications (Hay et al., 2006, Scharlemann et al., 2008). However, gaps within such time-series reduce the utility of these data sources for modeling and monitoring environmental phenomena, and gaps are particularly problematic within imagery of tropical and sub-tropical areas where persistent cloud-cover can obscure portions of the landscape seasonally or throughout the year. Gaps within fine temporal resolution time-series such as those derived from NASA’s Moderate Resolution Imaging Spectrometer (MODIS) imagery have been partially filled through the creation of products that summarize daily data into multi-day composites (e.g., 8- or 16-day). However, in the cloudiest of areas even composite products often contain problematic gaps, and these gaps take on added significance as they tend to occur in areas (e.g., equatorial Africa or the Amazon basin) for which few alternative geospatial datasets exist for characterizing dynamic landscape processes.

Our goals in this research were to develop a data-driven gap-filling methodology that (1) balances the need for accuracy with the computational efficiency necessary for feasible application to continental-scale time-series, (2) uses both spatial and temporal information within the data time-series to fill the gap pixels, (3) requires no ancillary datasets such as land cover products or digital elevation models to model missing pixel values, and (4) provides a standardized yet flexible approach that is applicable to a wide range of datasets. Among these goals, the first was most relevant to the wider remote sensing community as the large data volume associated with continental-scale time-series limits the utility of mathematically complex (e.g., geostatistical) algorithms for rapid gap-filling. Expected ancillary benefits of a conceptually simple approach include increased accessibility to a wider audience of potential image time-series users, as well as ease of adaptation of the developed methods for use with new datasets.

The gap-filling approach ultimately developed in this research is predicated on using both neighboring (non-gap) data and data from other time periods (i.e., calendar date or multi-year summary datasets) to fill gaps within image time-series. Our underlying hypothesis was that spatial and temporal autocorrelation inherent within longitudinal imagery archives can be leveraged to gap-fill remotely sensed data products. We developed and tested the gap-filling methodology using the MODIS Enhanced Vegetation Index (EVI) and Land Surface Temperature (LST) 1 km products, acquired for the African continent, from 2000–2012, with an 8-day temporal resolution. These data products were selected for eventual use in modeling malaria risk in Africa, but they are potentially useful for many research endeavors given their widespread utility. In particular, LST is correlated with air temperature (Mildrexler et al., 2011) and EVI is useful as a proxy (albeit lagged in time) for moisture in Africa (Jamali et al., 2011). Africa was selected as our study area because substantial portions of the continent experience widespread seasonal cloud cover, making this both an ideal region to test the methodology and an area in need of gap-filled products. Furthermore, processing time-series data for the whole of Africa presents a rigorous computational test for the presented gap-filling method.

## 2 Background

Numerous gap-filling approaches have been developed for modeling erroneous or missing data caused by clouds, shadows, or sensor malfunctions. These approaches can be roughly divided into the following categories: (1) methods that rely on spatial information, (2) methods based on temporal information available within an image time-series, and (3) methods that include both spatial and temporal information in the gap-filling process. Examples exist within each of these categories that include ancillary information, such as imagery from another sensor, a digital elevation model, or a classified land cover dataset, within the modeling process.

### 2.1 Spatial gap-filling approaches

Geostatistical approaches such as kriging have long been utilized for gap-filling imagery using the information present within surrounding (non-gap) pixels to interpolate missing data (e.g., Addink, 1999, Rossi et al., 1994). Introducing a second, gap-free dataset (e.g., an image from the same sensor acquired for the area of interest on a different date) enables gap-filling using cokriging techniques (Zhang et al., 2007, Zhang et al., 2009) as well as gap-filling approaches predicated on image segmentation (Bédard et al., 2008, Maxwell, 2004, Maxwell et al., 2007). Using data from an alternative date is also the technique underlying the novel Neighborhood Similar Pixel Interpolator method for filling gaps in Landsat ETM+ imagery developed by Chen et al. (2011), which was later augmented to include geostatistical theory by Zhu et al. (2012).

### 2.2 Temporal gap-filling approaches

The second category of gap-filling approaches relies on modeling missing pixel values using values associated with the missing pixel from different points in time, and a comparison of temporal approaches is provided in an informative review by Kandasamy et al. (2012). Jönsson and Eklundh (2004) made an important contribution to temporal approaches by developing the TIMESAT software package, which contains built-in asymmetric Gaussian and Savitzky–Golay filters for smoothing time-series data as a means of estimating missing data. Notable examples of temporal gap-filling applications include approaches for gap-filling MODIS Leaf Area Index (LAI) data (Gao et al., 2008) and NDVI derived from AVHRR data (Roerink et al., 2000). More recently Verger et al. (2013) developed the Consistent Adjustment of the Climatology to Actual Observations approach for increasing the accuracy of temporal interpolations of missing LAI data derived from AVHRR imagery by including climatological data within the model.

### 2.3 Spatio-temporal gap-filling approaches

Several spatio-temporal gap-filling approaches have been developed that utilize multi-step modeling approaches whereby the algorithm fills missing values using an alternating sequence of purely spatial or temporal steps. Kang et al. (2005) developed such an approach for gap-filling ecosystem metrics (i.e., fPAR, LAI, and net photosynthesis) modeled from MODIS data using simple spatial interpolation within land cover classes. If no cloud-free pixels were found within a 5 by 5 pixel window, the algorithm used temporal interpolation to fill the pixel using data from earlier and later dates. Borak and Jasinski (2009) later used a modified version of the Kang et al. (2005) approach when gap-filling MODIS LAI for a large portion of North America. Gafurov and Bárdossy (2009) also developed a stepped approach for gap-filling the MODIS snow cover product, but unlike the Kang et al. (2005) approach the algorithm developed by these authors prioritizes temporal gap-filling models and also includes a step that incorporates pixel elevation. More recently Poggio et al. (2012) developed an innovative method for gap-filling MODIS EVI data that utilizes a hybrid Generalized Additive Model (GAM) – geostatistical space–time model to model missing pixel values using spatial (latitude, longitude and elevation) and temporal (date of year) information as model covariates.

## 3 Materials and methods

**Fig. 1:**
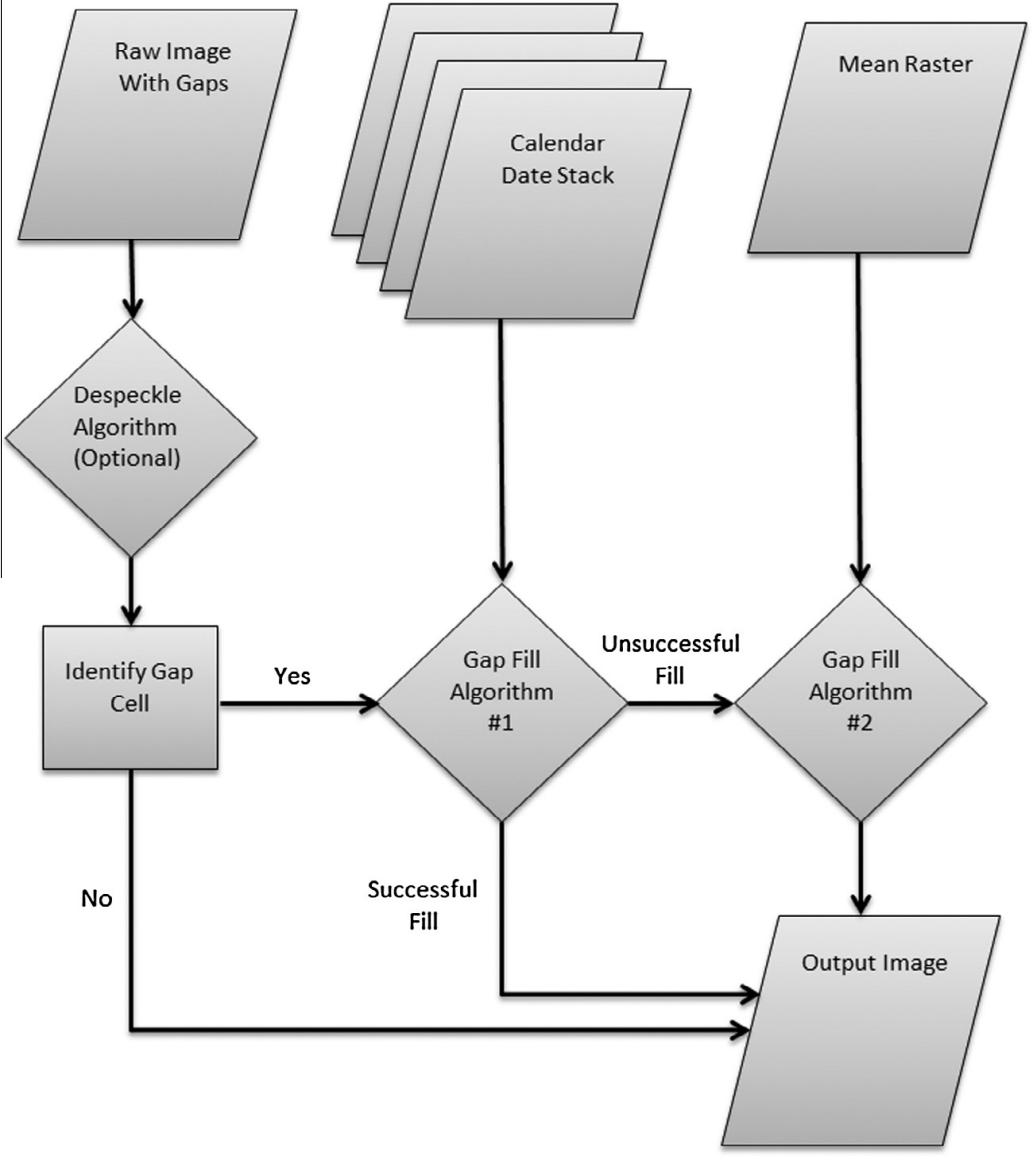
Overview of the generalized gap-filling model.

From our review of existing gap-filling methodologies we identified the Chen et al. (2011) approach as the most promising starting point for gap-filling the MODIS time-series of Africa due to its relative simplicity and computational efficiency. The immediate challenge in adapting this approach was to develop a fully operational algorithm capable of processing time-series data at a continental scale within a several-month time frame. Given these time constraints and the data volume of the project (i.e., nearly a terabyte in size) we ultimately developed two complementary algorithms that fill gaps by utilizing ratios from neighboring (non-gap) pixels derived at two points in time, similar to Chen et al. (2011), but modified for use with single-banded MODIS time-series to increase processing speed. The approach (Fig. 1 – explained in detail below) we develop (1) ingests raw images, (2) finds gap pixels that may first be identified using a despeckling algorithm, (3) fills some pixels using an algorithm that relies on calendar data imagery, and (4) fills the remaining gap pixels using a second algorithm that runs much faster by leveraging processing already used to fill adjacent gaps. Our gap-filling approach produces three output datasets for each image within a time-series: (1) a gap-filled image, (2) a flag image identifying the algorithm (if any) that was used for each pixel, and (3) a distance image quantifying the spatial lag between the filled pixel and the neighboring pixels used in the gap-filling model. We validated the approach by introducing and then filling artificial gaps within individual images, and we developed a technique for using the distance image to derive an estimated error associated with each filled pixel.

### 3.1 Datasets, pre-processing, and gap identification

The input datasets selected for this analysis were MODIS (1) MOD11A2 Land Surface Temperature (LST) 8-day composite data (Wan et al., 2002), and (2) MCD43B4 Bidirectional Reflectance Distribution Function (BRDF) – corrected 16-day composite data (Schaaf et al., 2002), from which Enhanced Vegetation Index (EVI) was derived using the equation defined in Huete et al. (1999). The MODIS LST dataset consists of both daytime and nighttime average temperatures aggregated, respectively, from the descending and ascending paths of the NASA Terra Satellite. The BRDF dataset contains 16-day products, with overlapping temporal windows that result in an 8-day temporal resolution, which were derived from data collected by the MODIS sensors on both the Aqua or Terra satellites.

The MODIS data were collected on a per-tile basis and then merged using the MODIS reprojection tool (Dwyer and Schmidt, 2006) to create seamless mosaics for all of Africa. A total of 42 tiles were required to cover the continent for each image date (i.e., the day of the year corresponding to the center of the composite temporal window). The BRDF mosaics each consisted of seven spectral bands, three of which were needed to derive the EVI, and mosaics were created for each of these bands prior to deriving the EVI for each image date. The resulting data archives consisted of 594 EVI mosaics (from day 049, 2000 to 361, 2012), and 590 LST-day and LST-night mosaics (from day 065, 2000 to 361, 2012). Temporal mean and standard deviation images were derived on a per-pixel basis from the full mosaic archives for each of the three variables for subsequent use in the gap filling algorithms. Producing images of summary statistics was also useful for identifying pixels that never contain usable data (e.g., ocean pixels) that could be ignored in the gap-filling procedures, thus reducing run-time.

The initial step in the gap filling process was to identify gap pixels in need of filling through the use of a despeckling algorithm, which is a processing step that need only be used if corresponding datasets describing pixel-level data quality do not exist. While MODIS products have associated quality assurance datasets useful for identify potential gaps, we developed a generic gap-finding approach to demonstrate the potential utility of our gap filling approach for a wide range of remotely sensed products. Gaps were identified by finding all pixels that contained a no-data or otherwise unacceptable value within the input mosaic that corresponded to usable pixels within the mean image, thus indicating that the pixel in question contained usable data on other dates. Unacceptable pixel values were identified by calculating a *z*-score for each pixel based on the mean and standard deviation images, and then searching for any pixel with an absolute *z*-score exceeding a user-defined threshold (we used 2.58, which corresponds to the 0.99 confidence interval, see supplemental information for more details). When such a pixel was found we examined neighboring pixels (we used a neighborhood size of 40 to 80 pixels) to determine if they were similarly unusual with respect to the mean value of the pixel. If the original *z*-score was beyond a second user-defined threshold (we used ±0.2) from the median neighborhood *z*-score, or if too few neighboring pixels were found within a user-defined search radius (we used 10 km), the original pixel was reclassified as a gap. In practice, pixels removed by the despeckling algorithm typically represent approximately 5% of gap pixels or 0.5% of all usable pixels present in the final output images.

Based on the results of the gap identification process the flag image was modified to indicate whether pixels were (1) a no-data pixel that should be ignored in subsequent processing, (2) a usable raw value that could be passed directly through to the final output (and is suitable for use in the gap-filling models), or (3) a gap to be filled. A preliminary analysis of the raw imagery mosaics indicated that, on average, approximately 5–15% of the pixels within an image were gaps in need of filling (Table 1).

**Table 1:** The mean and standard deviation percentages of gap pixels within the full Africa mosaics as calculated from the full imagery time-series (e.g., approximately 15% of a typical EVI mosaic consists of gap pixels).

Dataset	Proportion of missing pixels per image (%)	
	Mean	Standard deviation
EVI	14.77	5.93
LST day	5.25	2.28
LST night	8.51	3.28

### 3.2 Filling algorithm 1

**Fig. 2:**
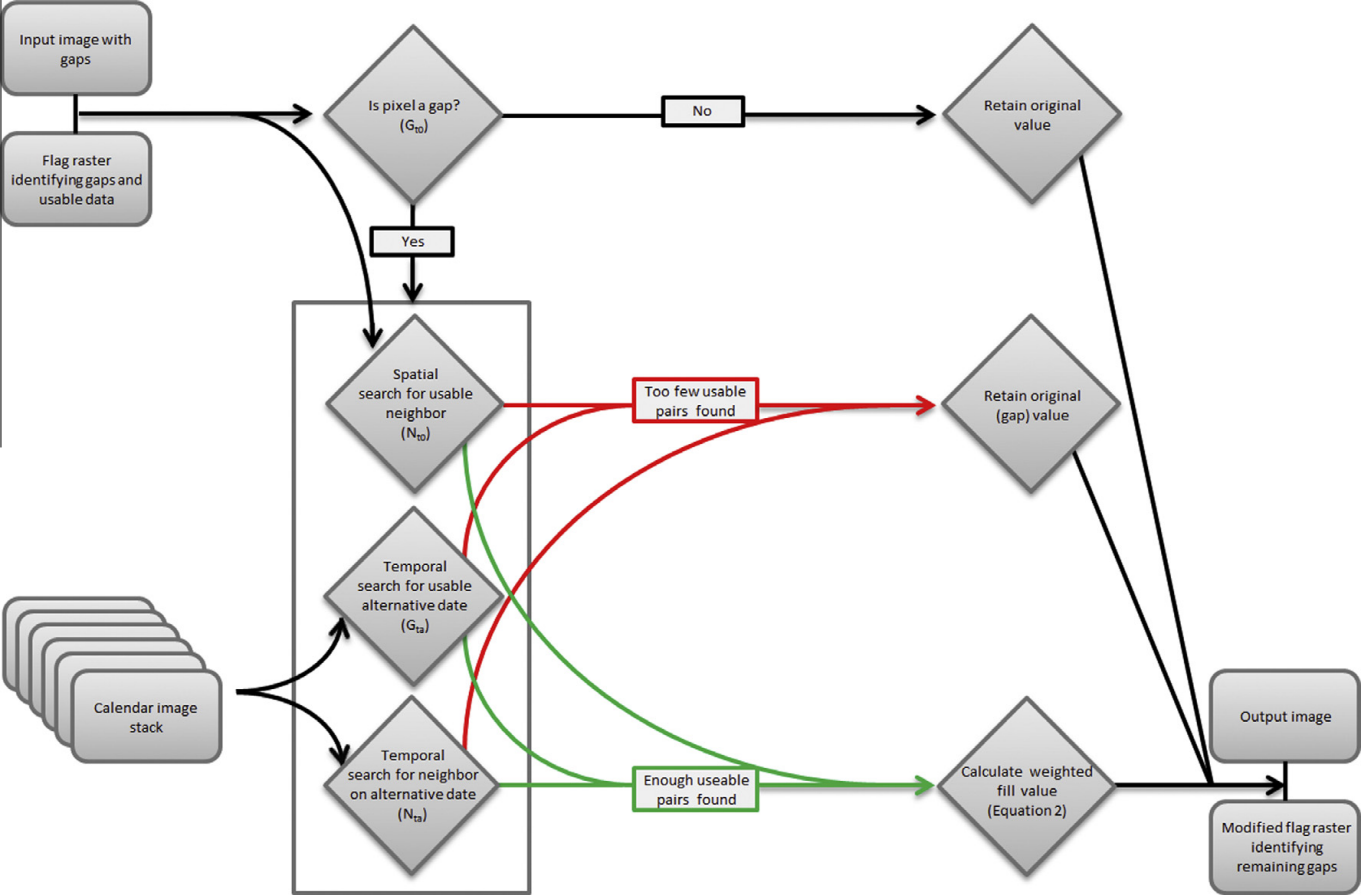
A conceptual diagram for the A1 algorithm.

The first gap-filling algorithm (Fig. 2) was conceptually similar to the approach used by Chen et al. (2011) for filling stripes within Landsat ETM+ imagery caused by the sensor malfunction that occurred in 2003. This approach first identifies neighboring pixels with similar spectral properties to a gap pixel, based on an image from another point in time, and then creates ratios (per-band) using values from the neighboring pixels to characterize the difference between the alternative time and the time of the gap image. Missing pixels can then be filled using the associated pixel value from the alternative time image, modified slightly based on the mean distance-weighted ratios from multiple neighboring pixels. The most significant modifications necessary for adapting the Chen et al. (2011) approach to our MODIS time-series relate to filling large gaps caused by clouds rather than the comparatively narrow, linear gaps produced by the Landsat ETM+ sensor malfunction. To fill gaps of larger sizes effectively we implemented an outward searching approach for finding neighboring pixels capable of searching much farther than the 17 by 17 pixel maximum window used by Chen et al. (2011). To increase the likelihood of finding usable neighboring pixels we did not restrict the search to only spectrally similar pixels for use as neighbor ratios. Instead, we utilized the temporal information available in the time-series and used only calendar dates (i.e., the same date on a different year) to preserve the underlying seasonal landscape patterns. This was considered a reasonable modification to the Chen et al. (2011) model because, unlike Landsat ETM+ pixels, most 1 km MODIS pixels contain a mixture of land cover types, thereby reducing the importance of spectral similarity within this modeling structure.

The first step in algorithm 1 (hereafter referred to as A1) for an unfilled image was to assemble a temporary image stack of all calendar dates from the image time-series. The algorithm then searched through the flag array for the unfilled image (referred to using the subscript *t*0 to signify the initial time period) to find pixels identified as gaps. When a gap-pixel was found, A1 searched the calendar date stack for any image that contained a usable value for the gap pixel (i.e., one with matching coordinates from a calendar date), starting with the preceding year (year −1) before proceeding to calendar dates from more distant years, searching both forwards and backwards in time (e.g., year +1, year −2, year +2, and so on). If a usable value for the gap pixel in the unfilled image (*G_t_*_0_) was found in an alternative year (*G_ta_*) the algorithm then searched outward from the gap pixel for neighboring pixels with usable values that were present in both the unfilled image (*N_t_*_0_) and the calendar date image (*N_ta_*). When an acceptable neighboring pair was found, the *G_ta_*, *N_t_*_0_, and *N_ta_* values, along with the spatial distance between *G* and *N* and the temporal distance between *t*0 and *ta* were used to calculate an weighted fill value (*F_i_*) (Eq. (1)), which was stored in a list along with the ratio of *N_t_*_0_, to *N_ta_* and the weight associated with the fill value (Eq. (2)). A simple inverse distance weighting (i.e., 1/distance ∗ 1/time) approach was applied at this stage to increase the contribution to the final fill value of the neighboring pixels that were closest in space and time. A full list of abbreviations used in equations within this paper can be found in Table 2.

(1) $$F_{i}=G_{\mathrm{ta}}\times \frac{N_{t0}}{N_{\mathrm{ta}}}\times \frac{1}{D}\times \frac{1}{\left|\mathrm{ta}-t0\right|}$$

(2) $$W_{i}=\frac{1}{D}\times \frac{1}{\left|\mathrm{ta}-t0\right|}$$

**Table 2:** List of abbreviations used in equations one through ten.

Abbreviations	Description
*F_i_*	The weighted fill value produced using A1
*G_t0_*	The final, modeled pixel value that replaces the gap at the initial time
*G_ta_*	The value of the gap pixel at the calendar (alternate) date
*N_t0_*	The value of the neighboring pixel at the initial time
*N_ta_*	The value of the neighboring pixel at the calendar (alternate) date
*W_i_*	The distance weight associated with the weighted fill value
*D*	The Euclidian distance between the gap pixel and the non-gap neighbor
*t0*	The signifier for the initial time period
*ta*	The signifier for the alternate time period (i.e., a calendar date)
*n*	The count of usable neighboring pixels
*F_p_*	The fill value associated with a single directional pass (*p*) of A2
*G_mean_*	The mean value for the gap pixel from the full 13-year imagery time series
*N_mean_*	The mean value for the neighboring pixel from the full 13-year imagery time series
*D_P_*	The distance associated with a directional pass (*p*) of the A2
*D_r_*	The residual distance “carried forward” for the neighboring pixel if that cell is a filled value
*D_A2_*	The distance value associated with the A2 gap fill
*Bias_D_*	The bias of the modeling error at distance (*D*)
*m_B_*	The slope (i.e., *b*1 coefficient) of the linear relationships between distance and modeling error bias
*b_B_*	The intercept (i.e., *b*0 coefficient) of the linear relationships between distance and modeling error bias
*StDev_D_*	The standard deviation of the modeling error at distance (*D*)
*m_s_*	The slope of the linear relationships between distance and modeling error standard deviation
*b_s_*	The intercept of the linear relationships between distance and modeling error standard deviation
*EE_CI_*	The estimated error for a modeled pixel within the defined confidence interval (*CI*)

The spatial search procedure spiraled outward from the gap pixel in a circular pattern based on a sorted distance table until either the threshold maximum number of neighbor pairs was found, or the maximum search radius was reached. If the maximum search radius was reached without the maximum threshold condition being met the algorithm then attempted to find a new *G_ta_* (i.e., a usable value for the gap pixel from a different calendar date) and, if one was found, the spatial search procedure was repeated on the associated calendar image. This process continued until the maximum threshold was reached or all calendar dates had been exhausted. If no calendar dates remained, but the maximum threshold had not been reached, a second threshold (the minimum number of ratio pairs acceptable for calculating a fill value using the A1 model) was compared to the number of usable neighbor pairs in the list. This minimum threshold allowed the algorithm to produce a fill value even when there were fewer usable neighbors than would be preferred. The maximum threshold, minimum threshold, and maximum search radius parameters were user-defined and provided a means of balancing model accuracy and processing time. In practice, we used values of 40 and 80 for the minimum and maximum thresholds, respectively, along with a maximum search radius of 3.6 km. The threshold values were selected based on a sensitivity analysis (see supplemental information) that demonstrated that using fewer ratio pairs produced unrealistic levels of spatial heterogeneity in the modeled output while searching for more ratio pairs and/or searching farther from the gap pixel increased run-times unnecessarily. However, these threshold values were calibrated only for use with the datasets and geographic location of this study and may require fine-tuning for other applications.

When the list of weighted fill values was complete a final, optional mechanism could be employed to reduce the impact of any anomalous pixels not identified by the error-detection (i.e., despeckling) procedure applied during pre-processing. In this procedure a user-defined proportion of the weighted fill list was removed based on the sorted *N_t_*_0_–*N_ta_* ratios so that the fill values associated with the most extreme (high and low) ratios were omitted from the final modeled estimate. The final step in A1 was to calculate the weighted mean value from all partial fill values remaining in the list (Eq. (3)). If the gap pixel was filled successfully the flag image was updated to reflect that the pixel was filled using A1 and the associated pixel value in the distance image was set to the mean spatial distance between each partial fill value pixel and the original gap pixel.

(3) $$G_{t0}=\frac{\sum_{1\ldots n}F}{\sum_{1\ldots n}W}$$

### 3.3 Filling algorithm 2

**Fig. 3:**
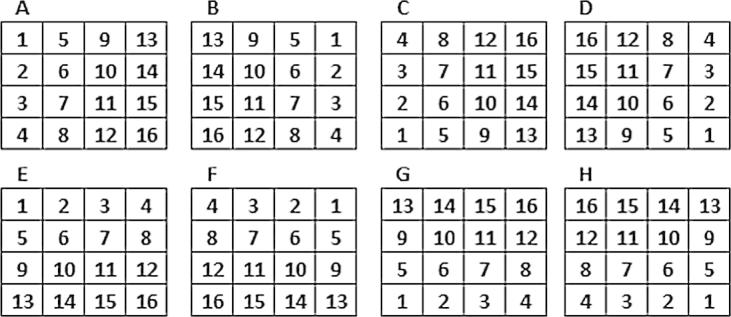
The processing order for pixels within a hypothetical four by four pixel gap for the eight passes of A2. Each of the either panels (A–H) represents a “directional pass” while the numbers indicate the order in which the pixels are processed.

During initial testing A1 was demonstrated to be adequate for filling small gaps within imagery, but this approach became computationally expensive for larger gaps due to the outward-searching algorithm. This issue was exacerbated by persistently cloudy areas that had few calendar dates with usable data or, in the most extreme case, pixels with no usable data for a given calendar date in any year within the time-series. As such it was necessary to develop an alternative algorithm (hereafter referred to as A2) to gap-fill continental scale time-series more quickly and without leaving any residual gaps. Conceptually, the gap-filling algorithms differ in three key ways. First, rather than relying on finding a usable neighboring pixel within both the unfilled image (*N_t_*_0_) and a calendar date image (*N_ta_*) the second approach used the value from the mean image (*N_mean_*) as the denominator in the ratio equation. Second, rather than looking outward (spatially and temporally) from a gap pixel (*G_t_*_0_) to find acceptable neighboring pairs, this A2 approach retained the ratio information from preceding pixels as it iterated through all pixels in the image, thereby carrying-forward information derived from the edge of a gap to subsequent gap pixels. Third, the alternative algorithm was run from eight directions (i.e., from each of the four corners of the image twice, once by row-column and again by column-row) (Fig. 3) and the median fill value from the eight passes was used as the final output. This directional approach ensures that pixels fill values from by A2 are informed by usable values from all edges of a multi-pixel gap. The “carry-forward” approach was accomplished by allowing modeled values from neighboring pixels to be used in the prediction of the current gap-filling value, including any neighboring pixels that preceded the current gap in the looping code architecture.

**Fig. 4:**
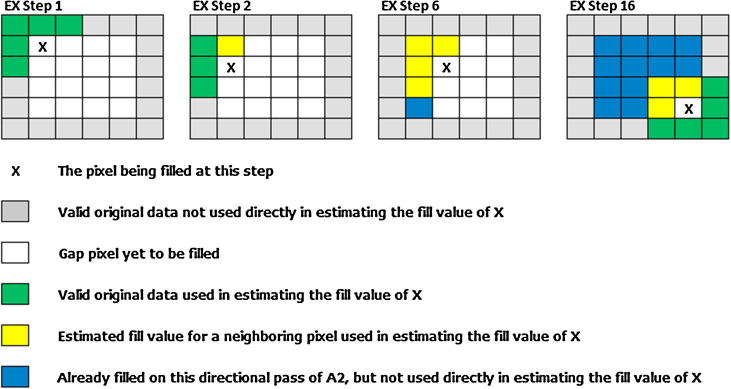
A hypothetical gap-filling example for a single pass (labeled “A” in Fig. 3) of A2.

A2 began by finding pixels in the flag array that were coded as a gap following the A1 algorithm, which indicated that A1 (if run) was unable to fill such pixels successfully. When a gap pixel (*G_t_*_0_) was encountered, per-pixel ratios were derived between any of the immediately surrounding pixels (*N_ta_*) containing usable data and the corresponding pixels in the mean image (*N_mean_*). Unlike A1, which relied on only pixels with usable raw data values, A2 utilized pixels containing (1) data usable in their raw form (i.e., non-gap pixels in the original imagery), (2) filled values computed using A1, and (3) values already filled from the current directional pass of A2 (i.e., pixels that were processed earlier in the looping structure of A2) (Fig. 4). All usable pixels (1…*n*) constitute the available data for filling the pixel using A2. The mean ratio from all available neighboring pixels was then multiplied by the value from the mean image for the original gap pixel (*G_mean_*) to produce a fill value for the gap (Eq. (4)). This derived fill value was then available for filling any neighboring gap pixel that had yet to be reached in this directional pass of A2.

(4) $$F_{p}=\frac{\sum_{1\ldots n}\left(G_{\mathrm{mean}}\times \frac{N_{t0}}{N_{\mathrm{mean}}}\right)}{n}$$

(5) $$G_{t0}=\mathrm{Median}(F_{1\ldots 8})$$

Algorithm A2 was applied from multiple directions to account for landscape heterogeneity, which was an important consideration given that the data spanned large areas. In the case of Africa, applying this algorithm using a single direction allowed, for example, gaps in savanna areas to be filled based solely on information gleaned from distant forests or *vice versa*. As such, we approached each gap pixel from multiple directions to “drag” the average conditions (i.e., the mean per-pixel ratio for that pixel) present from the nearest usable pixels from one direction. By taking multiple passes from different directions we approximated the outward-searching approach from A1 while greatly reducing the overall computation expense required to fill the gap.

As with A1, the flag image was modified to indicate which pixels were replaced by A2. Likewise, the distance image was amended to reflect the average distance from the newly filled pixel to the nearest pixels (in each of the eight directions) with usable values prior to running A2. Where the usable pixel was obtained from A1, the distance attributed to that fill was included in the A2 distance tabulation. Finally, where neither algorithm was able to fill a gap the resulting pixel was left as no-data. This was an exceedingly rare occurrence and tended to be restricted to pixels comprising small islands off the coast (i.e., pixels surrounded by large areas of no-data values in the ocean) that lacked any usable neighbors. These unfilled areas typically represented less than 0.01% of all gap pixels present within the resulting gap-filled datasets. The mathematical approach underlying the distance calculation in A2 is defined in Eqs. (6), (7).

(6) $$D_{P}=\frac{\sum_{1\ldots n}(D+D_{r})}{n}$$

(7) $$D_{A2}=\frac{\sum_{1\ldots 8}D_{p}}{8}$$

The variable *D_p_* denoted the distance associated with the current directional pass (1–8), *D* was the distance to the neighboring pixel (either 1.0 or 1.414) containing raw or previously modeled data, *D_r_* was the residual distance associated with the neighboring pixel that was 0.0 for raw data or the distance calculated in the filling process (using either A1 or A2) for that pixel, and *n* was the count of viable neighboring pixels on this pass. The final distance *D*_A2_ was then calculated as the mean of the eight directional passes. Note that because the distance values associated with A2 were calculated using information from multiple directions (i.e., from both the near and far sides of the gap that a pixel falls within) this metric reflected the effective size of the gap that the missing pixel fell within.

### 3.4 Calculating model uncertainty

Uncertainty associated with modeled pixel values has potential implications for downstream users of gap-filled imagery as a source of error and/or for incorporating the uncertainty within subsequent models. The uncertainty associated with gap-filled results for both algorithms was calculated by introducing artificial gaps into raw imagery and then comparing model outputs to known values. The introduced gaps were (1) distributed regularly to span a wide range of land cover types and (2) of varying sizes to assess the relationship between fill distance and model accuracy. After running the gap-filling algorithms on the input layer containing introduced gaps, we derived a table with the following information for all introduced gap pixels: the measured (i.e., original) pixel value, the filled value, the error (modeled minus measured), the distance value, and the flag value indicating the applied filling algorithm. We then divided the pixels based on the flag, subdivided the resulting groups into classes based on distance, and conducted the following analysis for both A1 and A2. Within each distance class the error mean and standard error were estimated as indicators of bias and error variability, respectively. To estimate these metrics we derived simple statistical models to predict both error bias and standard deviation as a linear function of fill distance (Eqs. (8), (9)) using the empirically derived (1) slope (*m_B_*) and intercept (*b_B_*) for the relationship between distance (*D*) and bias, and (2) slope (*m_S_*) and intercept (*b_S_*) for the relationship between distance (*D*) and error standard deviation. Using these parameters we calculated a final Estimated Error (*EE*), which represented the modeled uncertainty for a given confidence interval (Eq. (10)). To assess the maximum potential error associated with a gap fill we also included a constant term in the EE equation (in this case 1.96) that provided an estimate of uncertainty for the prediction. That is, by using the constant term 1.96 we can say that the fill value predicted for a given pixel will be within ±EE with 95% confidence. Also note that this approach for quantifying uncertainty has the added benefit of producing estimates in units of the variable being modeled.

(8) $${\mathrm{Bias}}_{D}=m_{B}\times D+b_{B}$$

(9) $${\mathrm{StDev}}_{D}=m_{s}\times D+b_{s}$$

(10) $${\mathrm{EE}}_{95\%}=|{\mathrm{Bias}}_{D}|+1.96\times {\mathrm{StDev}}_{D}$$

### 3.5 Model validation

**Fig. 5:**
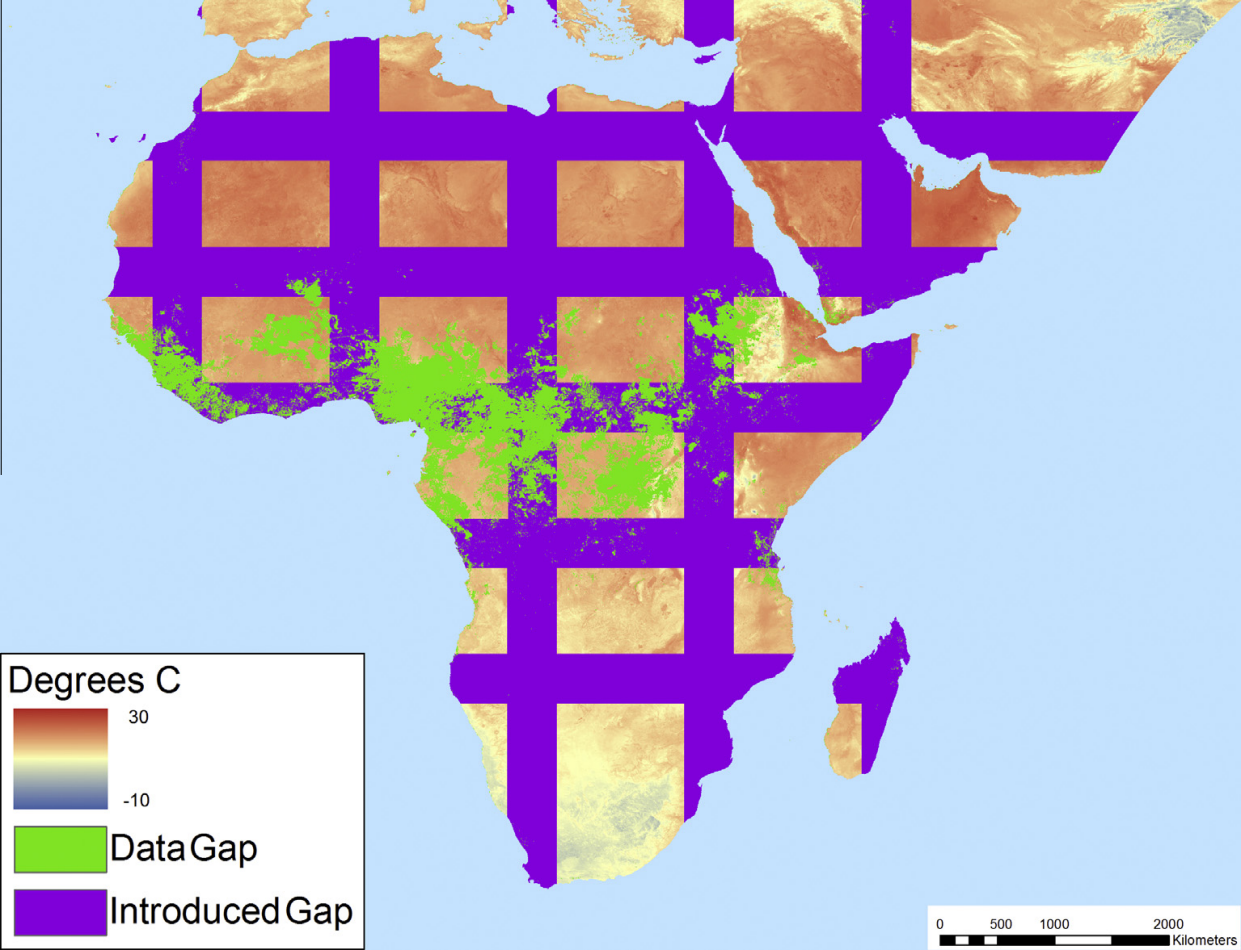
Example of 500 km validation stripes introduced within an LST image mosaic.

**Fig. 6:**
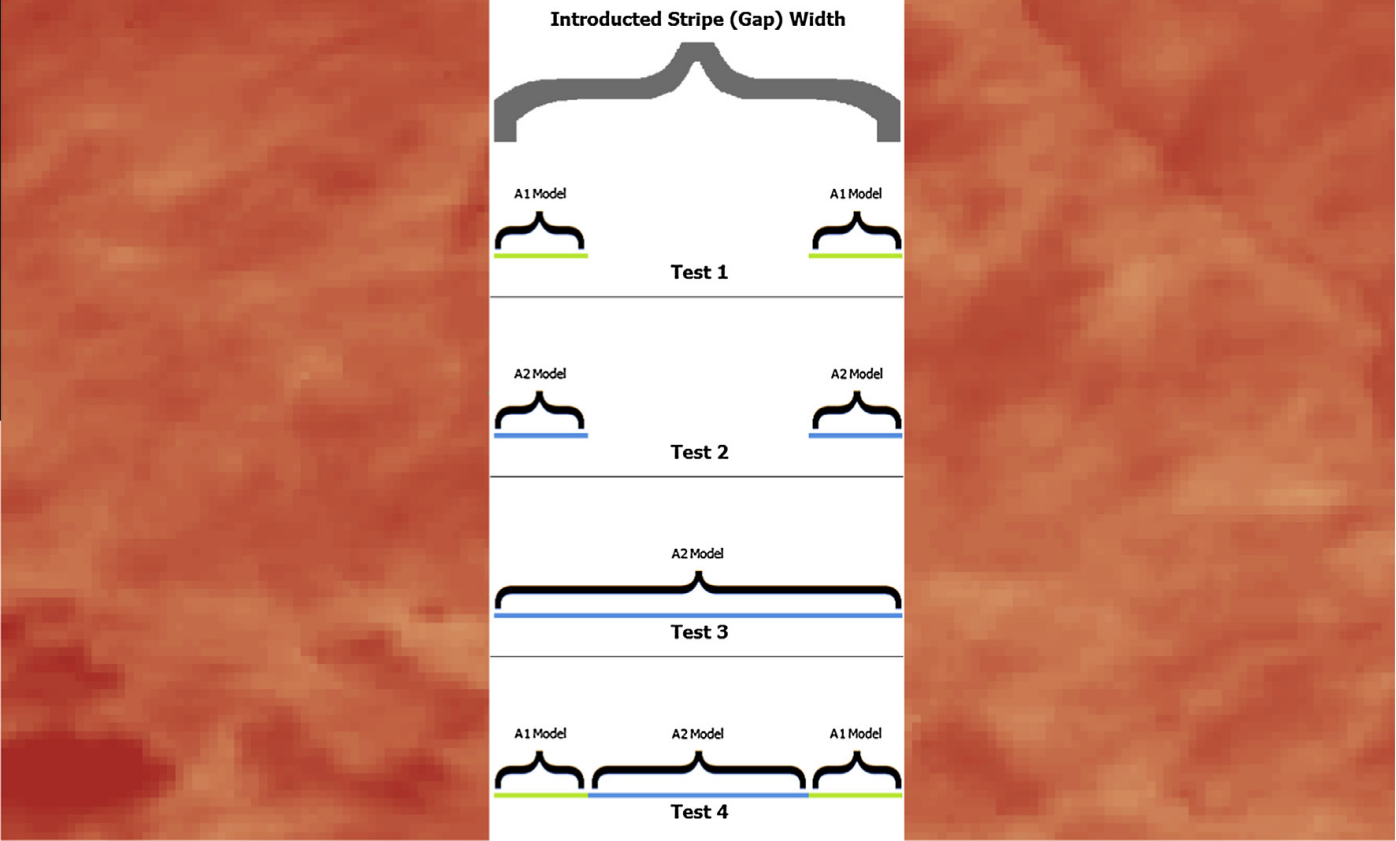
The 4-test validation process for assessing the accuracy of the gap filling procedure and comparing results from A1 and A2 models.

To assess the accuracy of the model results thoroughly we introduced stripes within the image (Fig. 5) at widths of 25 km and 500 km to match, respectively, the average and maximum gap sizes found in typical images. The striping approach ensured that we tested the accuracy of the models over all major land cover types and the different introduced gap widths provided estimates of expected accuracy in both the normal and worst case scenarios (i.e., all gaps being approximately equal to the maximum gap size found in a standard image). Note that a striping approach was utilized in lieu of alternatively shaped introduced gaps (e.g., irregular gaps representative of clouds) as this technique maximized the distances associated with the filling procedure because there were no usable pixels along the major axis of the stripe or stray usable pixels within gaps. We then employed a 4-test validation process (Fig. 6) to compare and contrast the accuracy of the A1 and A2 model results. The purpose of tests one and two was to compare A1 and A2 directly on the same set of pixels as a means of assessing the relative accuracy of each when gap filling all cells located close to usable neighbors using each algorithm exclusively. Test three extends test two to include all gap pixels, thereby allowing the decline in accuracy of the A2 model as a consequence of increasing distance to be assessed directly. Test four then assessed the hybrid model that uses both algorithms which, when compared to the test three results, provides a means of assessing whether the A1 algorithm is worth the additional computational cost associated with running it. Furthermore, the fourth test is useful for ensuring that potential error propagation resulting from using A1 results as inputs for A2 did not negatively impact the resulting fill values. Lastly, to test the processing times associated with A1, A2, and the optimized composite model (gleaned from the sensitivity analysis) a preliminary analysis was conducted, the results of which (Table 3) illustrate the advantage of utilizing A2 or the composite approach for gap filling the 1 km resolution images of Africa.

**Table 3:** A processing time test for comparing the A1, A2, and hybrid gap filling approaches. The comparison dataset was a single EVI mosaic, gap filling for all three tests was conducted using a single core on a desktop workstation, and all runtimes are in minutes. Note that the A1 approach was capped at a 100 km search radius and thus still utilized A2 to fill some gap pixels.

Gap filling model	A1 runtime	A2 runtime	Total runtime	% Gaps filled by A1	% Gaps filled by A2
A1 “only”	3587.0	15.4	3602.4	93.25	7.72
A2 only	0.0	29.6	29.6	0.0	99.96
Composite (A1 & A2)	158.5	22.8	181.3	41.36	58.6

## 4 Results

The core datasets resulting from this research are 8-day daytime LST, nighttime LST, and EVI products that were gap-filled to create spatially and temporally complete datasets for all of Africa from 2000 to 2012. Gap-filled results were produced for all dates within each of the three datasets (i.e., 1774 individual layers) except seven dates for which the raw mosaics were deemed too poor (i.e., incomplete) to gap-fill reliably. To illustrate the results we provided animations of the results (averaged to a monthly time-step) for each variable as additional files (EVI.m4v, LST_day.m4v, and LST_night.m4v).

### 4.1 Example results for a single image

**Fig. 7:**
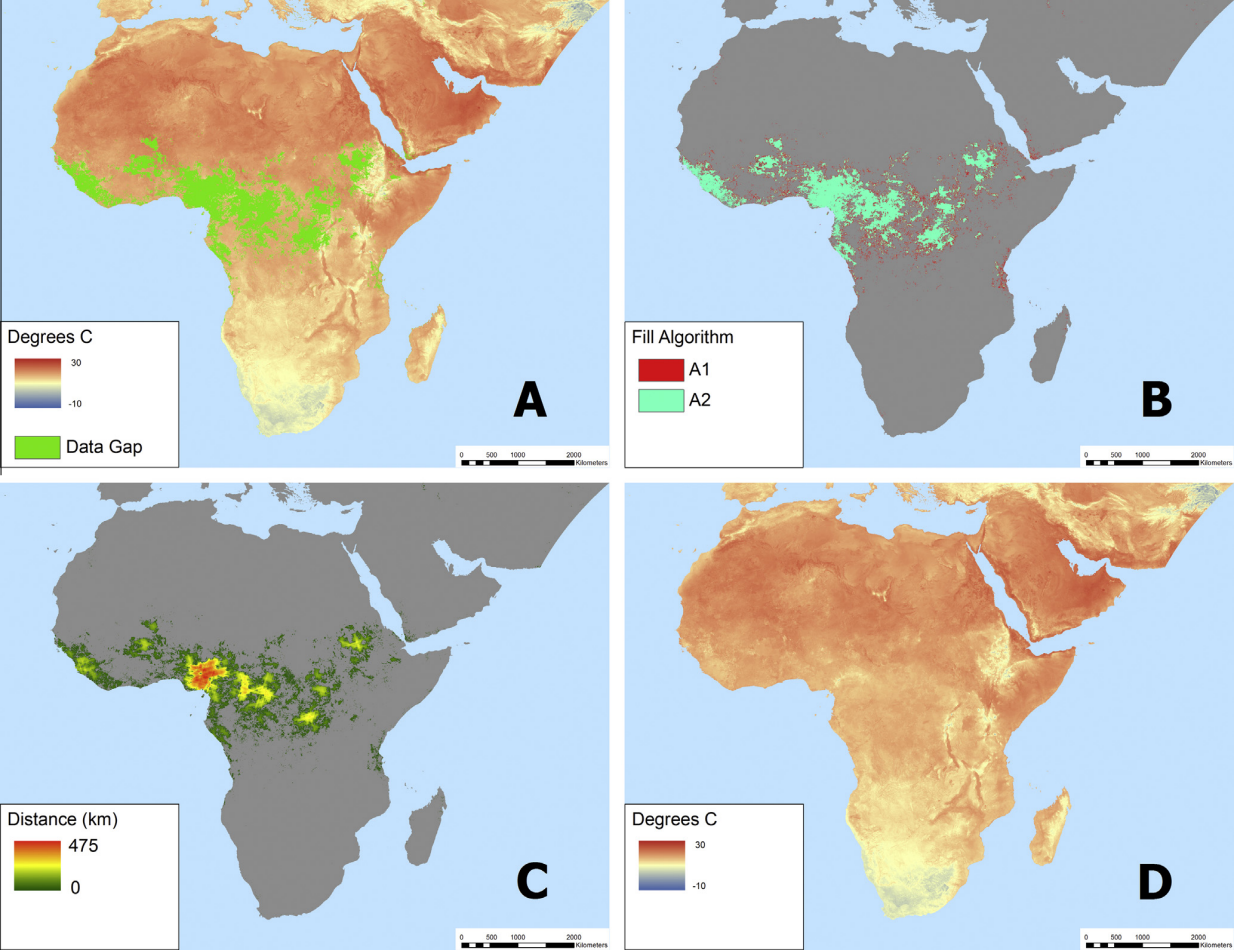
The input and output image layers associated with a single gap-filled result for the nighttime LST image from day 241, 2012. Map A shows the raw image mosaic, with green areas indicating missing data. The remaining maps show results from the modeling process, with Map B showing which model (A1 or A2) was used to fill each gap pixel (i.e., the flag image), Map C showing the distance associated with the gap filling procedure, and Map D showing the resulting gap-filled output.

**Fig. 8:**
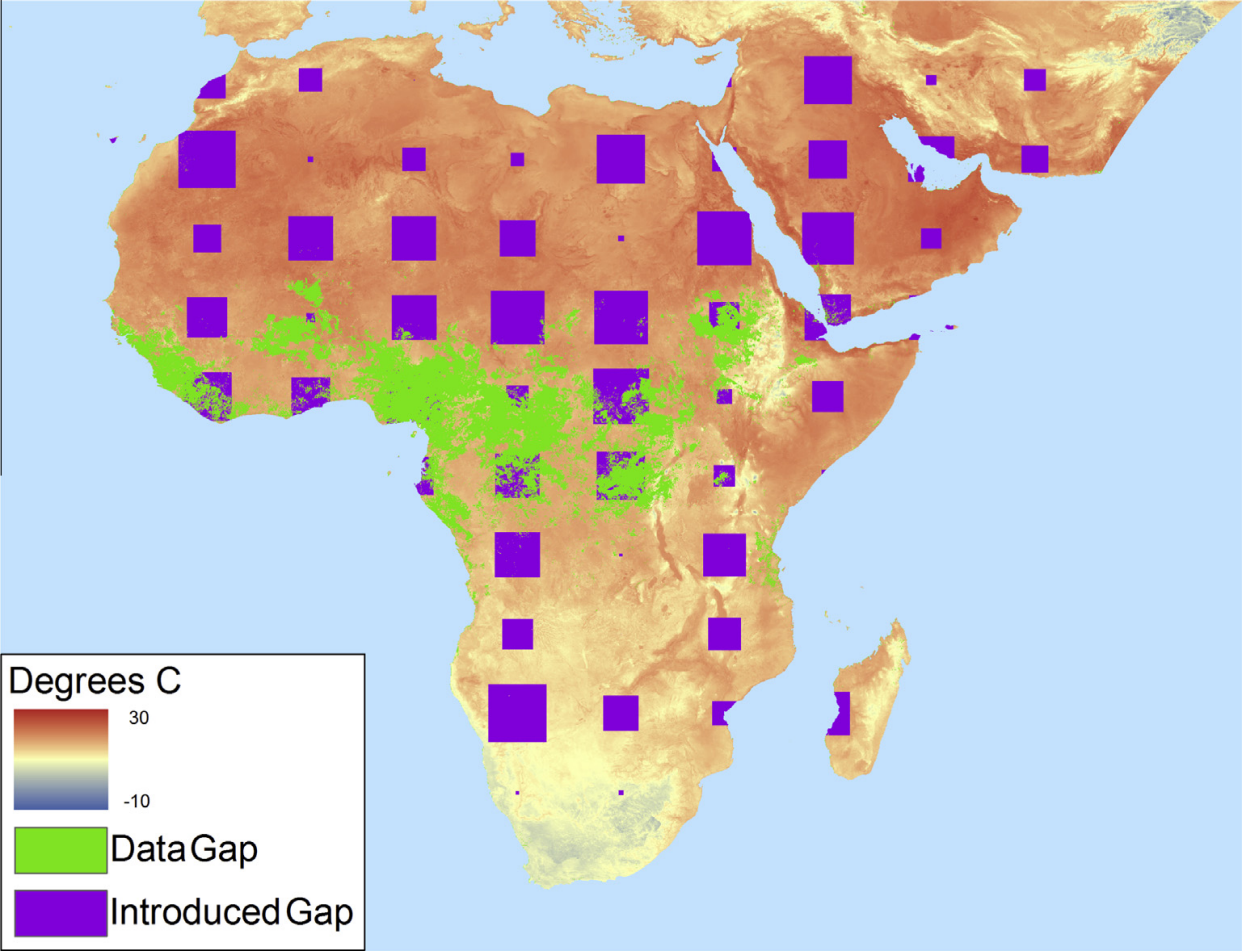
Introduced gaps of varying sizes used to model uncertainty in the gap filling process.

**Fig. 9:**
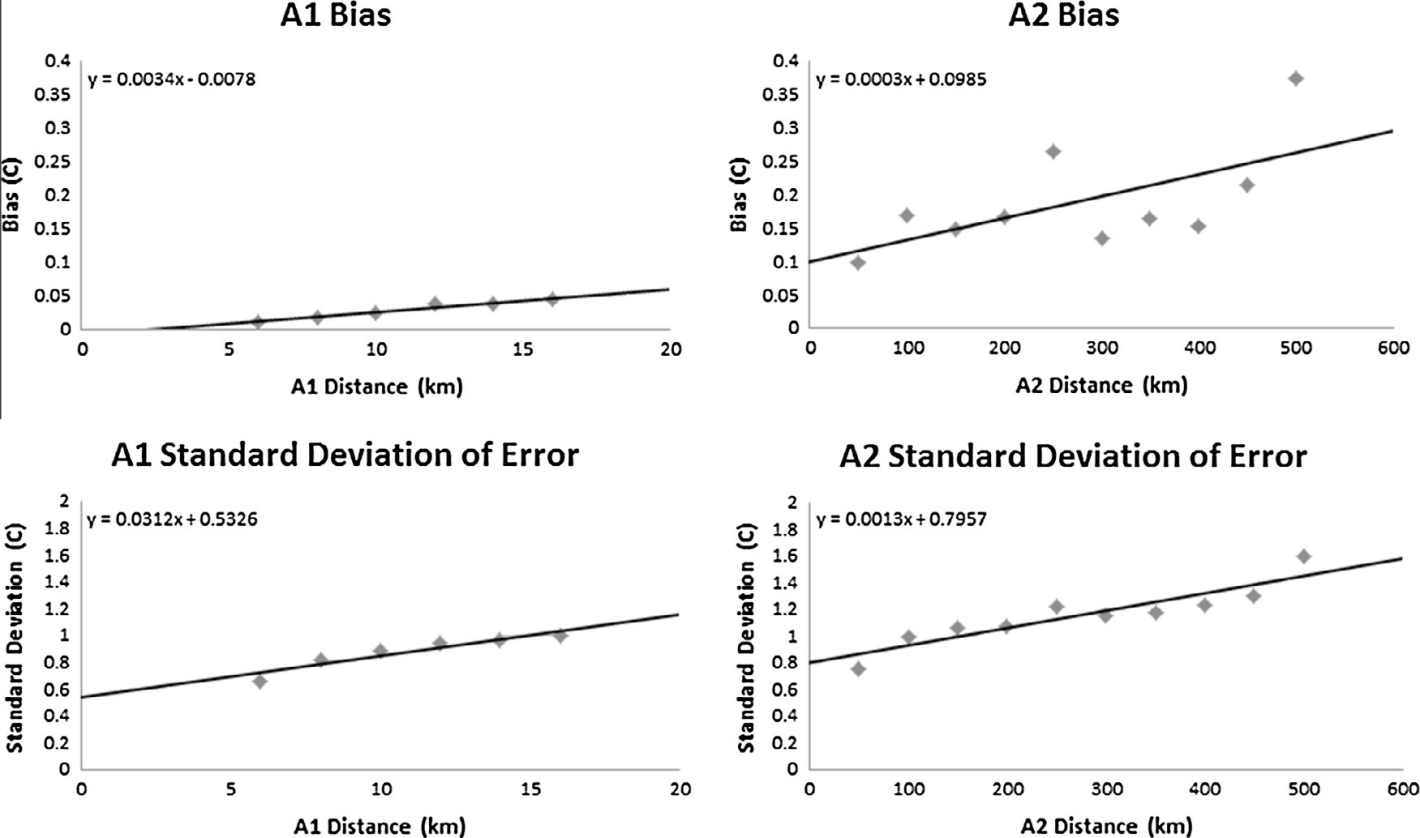
Bias and standard deviation of the gap-filled errors (i.e., modeled minus measured) for the introduced gap pixels. The equations shown on these plots were applied subsequently to the original filled data (according to the fill algorithm used) to produce the final estimated maximum error.

**Fig. 10:**
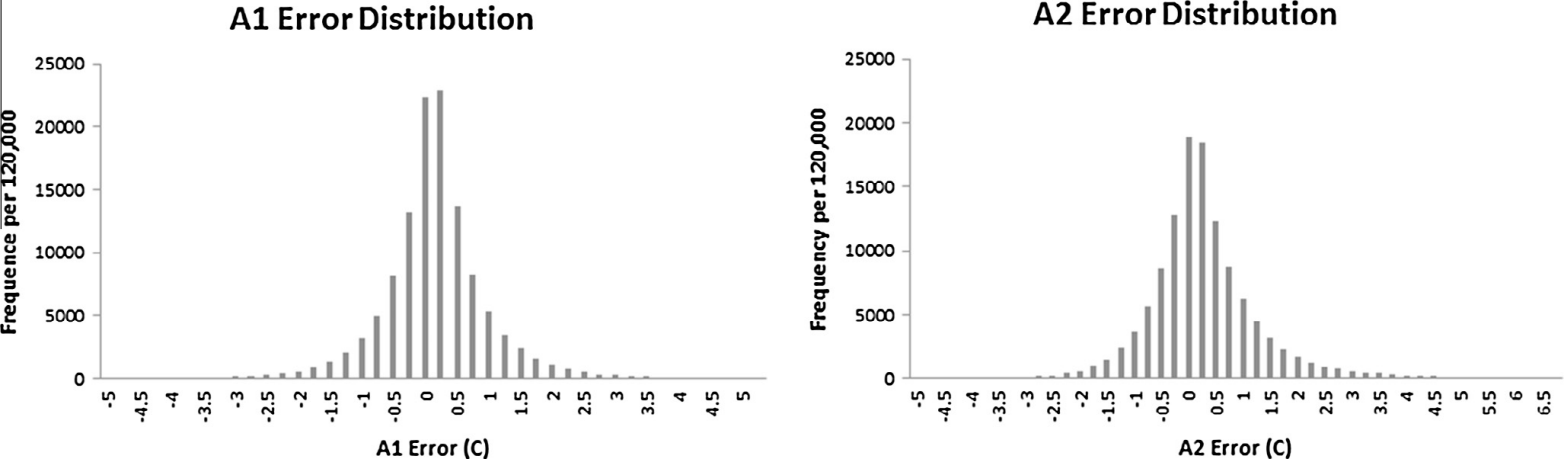
The distribution of model error (i.e., modeled minus measured) for a sample of 120,000 artificially created gap pixels from gaps of varying sizes.

**Fig. 11:**
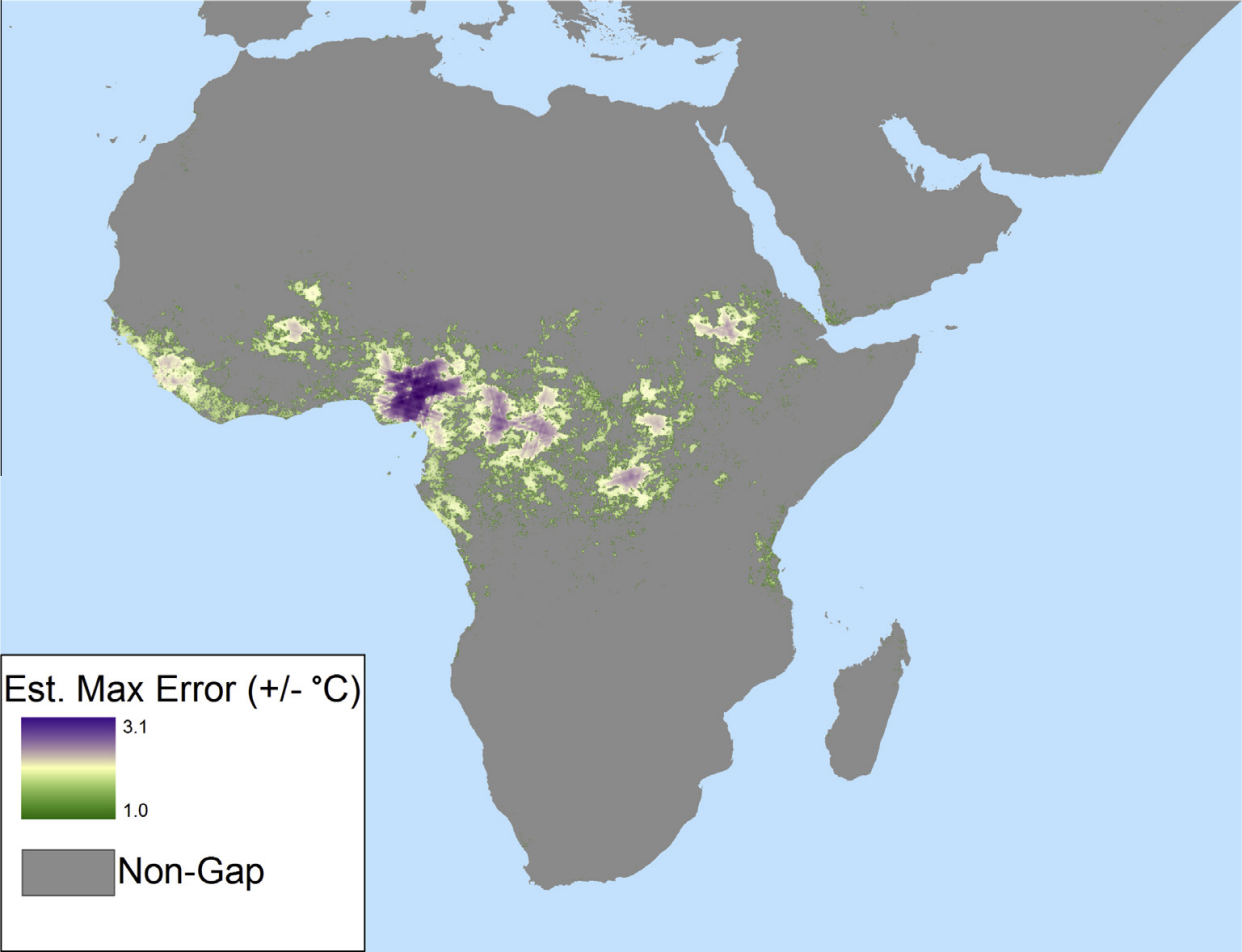
The map of estimated maximum error for the gap-filled output. Based on this product we can say with 95% confidence that filled gap pixels within the selected LST nighttime image are within (±) the number of degrees Celsius indicated on the map.

Given the volume of results (i.e., the output consists of multiple images for each of the 1767 image layers) we present only the input and output images associated with a single variable on a single date (nighttime LST from day 241, 2012) (Fig. 7). To derive the estimated maximum error image for this example LST image we first introduced artificial gaps of varying sizes throughout the image (Fig. 8). After extracting details (i.e., the original value, filled value, algorithm used, and distance) for a sample of 120,000 introduced gap pixels, we binned the pixels by distance classes to explore the intra-class model bias and standard deviation of the differences between the modeled and measured values (Fig. 9). Using these relationships we defined the bias and standard deviation (see Eqs. (9), (10), respectively) as a function of distance. Overall model bias was quite low (in the case of nighttime LST, only ∼0.25 °C with gap distances of 500 km), but this aspect of uncertainty was modeled separately so that a simple linear correction factor could be applied to output results if needed. Because the model errors have an approximately normal distribution (Fig. 10) we applied a coefficient associated with the 95% confidence interval (i.e., 1.96, see Eq. (10)) to produce our final estimate of maximum error for each filled pixel. Lastly, we applied this function back to the original gap filling output (e.g., the image shown in Fig. 7C) to produce the final uncertainty map (Fig. 11) that incorporates both aspects of uncertainty in a single image.

### 4.2 Validation results for all datasets

To assess model accuracy for each dataset we applied the four-test validation procedure (see Fig. 6) based on introducing gap stripes at the average and maximum distances typically seen in the input mosaics. For each of the datasets, five or six images were selected randomly for validation, and the results are shown in Table 4. Overall model accuracy was very high, with mean *R*^2^ values above 0.87 for all variables, even for the 500 km stripes (i.e., the worst case scenario where all introduced gaps in the image are as large as the maximum gap size found in typical images). The RMSE results are equally robust, with RMSE values for LST of 2.49 C or better, and the largest RMSE value for EVI being 0.037. As these largest RMSE values are associated with introduced stripes of 500 km, they represent an accuracy floor that all gap-filled images are very likely to exceed in this study (i.e., for the three variables for Africa).

**Table 4:** The validation results for the three gap-filled datasets (indicated by the type column). Random dates were selected for each of the datasets (indicated by year, day, and date columns), with mean values for each section shown in bold. Validation was conducted by introducing 25 km and 500 km stripes (indicated by the stripe width column), and four tests were run on each validation image following Fig. 6 (indicated by the test columns).

Year	Day	Date	Type	Stripe width	Test 1		Test 2		Test 3		Test 4	
					*R*^2^	RMSE	*R*^2^	RMSE	*R*^2^	RMSE	*R*^2^	RMSE
2001	145	May 25th	LST night	25	0.987	0.546	0.986	0.576	0.973	0.795	0.976	0.750
2004	65	Mar 5th	LST night	25	0.982	0.714	0.987	0.610	0.974	0.863	0.974	0.865
2006	177	Jun 26th	LST night	25	0.993	0.539	0.989	0.673	0.975	0.994	0.980	0.890
2011	321	Nov 17th	LST night	25	0.989	0.629	0.989	0.626	0.978	0.895	0.978	0.895
2012	241	Aug 28th	LST night	25	0.986	0.553	0.981	0.644	0.963	0.920	0.969	0.838
**Mean**			**LST night**	**25**	**0.987**	**0.596**	**0.986**	**0.626**	**0.973**	**0.893**	**0.975**	**0.848**

2001	145	May 25th	LST night	500	0.988	0.541	0.980	0.713	0.890	1.390	0.896	1.335
2004	65	Mar 5th	LST night	500	0.985	0.684	0.984	0.745	0.917	1.389	0.919	1.376
2006	177	Jun 26th	LST night	500	0.994	0.516	0.981	0.903	0.907	1.712	0.910	1.647
2011	321	Nov 17th	LST night	500	0.989	0.666	0.981	0.906	0.915	1.526	0.969	1.511
2012	241	Aug 28th	LST night	500	0.989	0.524	0.975	0.796	0.880	1.519	0.877	1.516
**Mean**			**LST night**	**500**	**0.989**	**0.586**	**0.980**	**0.813**	**0.902**	**1.507**	**0.914**	**1.477**

2001	129	May 9th	LST day	25	0.989	0.965	0.991	0.884	0.983	1.181	0.983	1.179
2005	257	Sep 14th	LST day	25	0.985	0.928	0.987	0.860	0.977	1.162	0.978	1.142
2005	9	Jan 9th	LST day	25	0.983	1.105	0.985	1.020	0.972	1.416	0.973	1.392
2006	73	Apr 14th	LST day	25	0.973	1.087	0.980	0.948	0.965	1.271	0.967	1.273
2007	145	May 25th	LST Day	25	0.988	1.016	0.990	0.925	0.983	1.214	0.983	1.204
**Mean**			**LST day**	**25**	**0.983**	**1.020**	**0.987**	**0.927**	**0.976**	**1.249**	**0.977**	**1.238**

2001	129	May 9th	LST day	500	0.990	0.934	0.988	1.045	0.943	1.953	0.944	1.942
2005	257	Sep 14th	LST day	500	0.986	0.892	0.978	1.117	0.898	2.189	0.900	2.167
2005	9	Jan 9th	LST day	500	0.986	1.049	0.980	1.313	0.894	2.455	0.895	2.429
2006	73	Apr 14th	LST day	500	0.979	1.023	0.978	1.051	0.888	2.024	0.890	2.003
2007	145	May 25th	LST day	500	0.989	0.946	0.988	1.012	0.942	1.983	0.942	1.976
**Mean**			**LST day**	**500**	**0.986**	**0.969**	**0.983**	**1.108**	**0.913**	**2.121**	**0.914**	**2.103**

2000	97	Apr 6th	EVI	25	0.987	0.016	0.986	0.017	0.978	0.022	0.979	0.022
2000	129	May 8th	EVI	25	0.984	0.017	0.985	0.016	0.976	0.021	0.977	0.021
2000	201	Jul 19th	EVI	25	0.983	0.013	0.978	0.015	0.967	0.019	0.969	0.018
2003	17	Jan 17th	EVI	25	0.983	0.017	0.984	0.016	0.975	0.022	0.976	0.022
2007	281	Oct 8th	EVI	25	0.984	0.017	0.983	0.018	0.975	0.024	0.976	0.023
2008	345	Dec 12th	EVI	25	0.979	0.017	0.978	0.017	0.969	0.022	0.970	0.022
**Mean**			**EVI**	**25**	**0.983**	**0.016**	**0.982**	**0.017**	**0.973**	**0.022**	**0.975**	**0.021**

2000	97	Apr 6th	EVI	500	0.988	0.016	0.984	0.018	0.919	0.033	0.918	0.033
2000	129	May 8th	EVI	500	0.986	0.016	0.982	0.019	0.925	0.033	0.925	0.033
2000	201	Jul 19th	EVI	500	0.983	0.013	0.978	0.015	0.899	0.030	0.896	0.030
2003	17	Jan 17th	EVI	500	0.984	0.015	0.978	0.018	0.893	0.037	0.893	0.037
2007	281	Oct 8th	EVI	500	0.987	0.016	0.983	0.019	0.892	0.035	0.895	0.036
2008	345	Dec 12th	EVI	500	0.982	0.015	0.973	0.019	0.920	0.037	0.920	0.037
**Mean**			**EVI**	**500**	**0.985**	**0.015**	**0.980**	**0.018**	**0.908**	**0.034**	**0.908**	**0.034**

## 5 Discussion

The original objective of this research was to adapt the model developed by Chen et al. (2011) and apply that adapted method to single-banded MODIS product time-series for Africa. We opted for this approach rather than a more mathematically sophisticated method to keep run-times low and retain a conceptually simple model that can easily be adapted for use with many time-series datasets. Furthermore, expending the additional coding effort and processing time required of such methods was challenging to justify as previous research has shown that more sophisticated techniques such as a geostatistical approaches are not necessarily superior to simpler methods for interpolating missing data within imagery (Lloyd and Atkinson, 2002). The A1 model represents the implementation of the modified version of the Chen et al. (2011) approach and the validation results illustrate the high accuracy this algorithm is capable of producing. In practice, however, the run-times associated with the A1 model were too long to feasibly process more than 1700 continental scale images at 1 km spatial resolution. Efforts to increase the efficiency of the A1 model resulted in some performance improvements (i.e., increases in processing speed), but ultimately the algorithm could not be made efficient enough to allow practical computation, as the outward searching algorithm did not lend itself well to a parallel computing architecture. As such, while A1 would be the preferred approach for processing smaller datasets (i.e., shallower time-series, smaller spatial extents, or coarser spatial resolutions), we chose to develop an alternative algorithm (A2) to create a more generally applicable framework for continental-scale processing.

The goal in developing A2 was to approximate the A1 approach of searching outward for neighboring ratio pairs, while continuing to produce highly accurate results, but in a much more computationally efficient manner. Conceptually, A1 and A2 differ in that A1 searches outward from a gap pixel for a usable neighbor while A2 “drags” values from the edge of the gap to each gap pixel. Using modeled values of neighboring pixels rather than relying on only usable raw values produces the leap in computational efficiency associated with A2 (see Table 3). This modification effectively recycles the computational cost already spent filling neighboring gaps, and it is particularly effective at reducing processing times for pixels within large gaps.

Two important and potentially problematic aspects of A2 are (1) the propagation of error from the A1 model when the A2 model incorporated A1 results as model input, and (2) the introduction of seasonal bias related to the use of the mean dataset as the source of the denominator values in the ratio pairs. Error propagation was taken into account by adding the residual distance (i.e., the distance associated with the A1 modeled output) to the A2 distance image and, more importantly, by modeling uncertainty independently for A1 and A2. The use of the mean dataset could have introduced seasonal bias if, for example, the annual occurrence of the rainy season coincided with spatial patterns in EVI or LST that were underrepresented in the mean images. Ultimately, however, the results stand for themselves as the A1 and A2 models produced very similar accuracies (i.e., the *R*^2^ values for tests one and two are ±0.01 for all variables). These findings indicate that, at least for the variables examined in this analysis, possible effects within the mean image related to land cover patterns and seasonally persistent cloud-cover did not reduce the accuracy of the A2 algorithm.

Our validation results show that A2 is nearly as accurate as A1 (see Table 4), but A2 runs much faster (typically in about 1/100th of the time) when gap-filling a typical EVI or LST mosaic for Africa. Ultimately, we opted to use both A1 and A2 in a composite approach (i.e., test four in Fig. 6) as (1) the algorithms were designed to be complementary since results from A1 were used as input data for the A2 model, and (2) we wanted to retain the favorable properties related to A1 when it was computationally reasonable to do so (e.g., when the gaps were small). Specifically, we were reluctant to abandon A1 as it is better equipped to incorporate intra-annual variability due to its use of calendar date imagery. Furthermore, by preferentially selecting calendar dates from years closer in time, A1 is at least theoretically able to account for some land cover changes, albeit only in serendipitous instances when both the gap and the calendar date image(s) from which the ratios are being drawn are from before or after the land cover change event. For example, if a land cover change occurred for a given pixel in the second year of a 13 year data series, and the A1 model was attempting to fill a missing value for that pixel found in year three, neighbor ratios created from years two and four to 13 would all reflect that change while neighbor ratios from year one would not.

By creating two algorithms that can be used independently or in conjunction, the presented gap-filling approach offers flexibility for balancing the accuracy of modeled results with data production times and/or the computational resources available. Furthermore, the gap-filling models each contain multiple user-defined thresholds that allow users to fine-tune the model parameters. For example, user-defined parameters of the A1 algorithm include the maximum search radius used to find neighboring ratio pairs, as well as the number of usable ratios required to calculate the resulting fill value. While the presence of modifiable parameters presents a slight challenge for users who wish to adapt this approach to new datasets, a preliminary sensitivity analysis starting with the values presented in the manuscript (and elaborated upon in the supplemental information) will allow users to balance run-times, given the nature of the time-series dataset, and the acceptable uncertainty of the results.

As with all modeled data products, some uncertainty is associated with the final output from our hybrid gap-filling procedure. To account for uncertainty we utilized an intensive sampling approach whereby we created a large sample (*n *= 120,000) of modeled pixel values, within artificial gaps of varying sizes (see Fig. 8), and distributed widely across the African continent. This approach enabled us to estimate the maximum error associated with each predicted gap pixel value while incorporating a large number of sample pixels from all major land cover types. The resulting images provide robust estimates of uncertainty in the units of the dataset being modeled (e.g., in degrees Celsius for the LST products), which allows the modeled uncertainty to be readily incorporated within subsequent analyses. However, neither our gap-filling method nor our uncertainty metric can account fully for error associated with land cover changes, a limitation that could potentially be addressed in future research via the inclusion of ancillary datasets. While the level of acceptable uncertainty will vary according to the specific, eventual use of the gap-filled product, the results of the nighttime LST example indicated (via RMSE, see Table 4) that the average error for a filled pixel (relative to the raw MODIS LST value) is at worst ∼1.5 °C and likely closer to ∼0.6 °C. These values are quite close to the 0.5 °C error associated with the raw MODIS LST (relative to *in situ* LST measurements) products as reported by Wan (Wan, 2008), which suggests that the combined RMSE for a resulting gap-filled nighttime LST images would range from approximately 1.1 to 2.0 °C relative to *in situ* measurements. An important caveat to this finding, however, is that LST is impacted by cloud cover (i.e., the underlying cause of most gap pixels), which means gap filled LST data most accurately represents “clear sky” LST conditions.

## 6 Conclusion

The novel gap-filling approach presented in this research represents an adaptation of existing techniques to create an operational method that is applicable to continental-scale image time-series. While our analysis was restricted to MODIS products, the described method could be readily adapted to a very wide variety of remotely sensed time-series, irrespective of the cause(s) of the missing data. Our method produces highly accurate results while utilizing a conceptually simple, computationally efficient algorithmic framework that leverages the wealth of empirical information present within large imagery time-series to fill missing pixels. This data-driven, spatio-temporal approach represents a departure from more commonly used, model-based approaches for gap-filling missing pixels. Additionally, our approach does not rely on ancillary datasets such as land cover class maps or digital elevation models that require acquisition of additional data and potentially introduce new sources of error to the modeling process (e.g., in cases where landcover is misclassified). Our method of estimating model error provides a means of characterizing model uncertainty for all gap-filled pixels in a format that can be readily passed along to downstream applications of the gap-filled datasets. Lastly, the use of two complementary algorithms, in conjunction with user-defined parameters inherent to the approach, offers the flexibility necessary to address real-world limitations associated with large data volumes and processing demands, limited computational infrastructure, and time-sensitive products.

## Funding sources

PWG is a Career Development Fellow (#K00669X) jointly funded by the UK Medical Research Council (MRC) and the UK Department for International Development (DFID) under the MRC/DFID Concordat agreement and also receives support from the Bill and Melinda Gates Foundation (#OPP1068048). These grants also support DW, SB, and BM.
