# Supplementary material for: An effective approach for gap-filling continental scale remotely sensed time-series
Source: ISPRS J Photogramm Remote Sens. 2014 Dec;98:106–18. doi: 10.1016/j.isprsjprs.2014.10.001 (PMC4308023; doi:10.1016/j.isprsjprs.2014.10.001)
Supplement: Supplementary data 1 [file mmc1.docx]

**Supplemental Information for User Defined Thresholds**

**S.1 Introduction**

The gap-filling algorithms presented in this research have a number of user-defined thresholds that enable end users to balance the accuracy of final modeled results, the desired degree of smoothing, and the computational cost associated with gap-filling a given dataset. This supplemental document contains the results of sensitivity analyses that were conducted to support the selection of these thresholds. While the reported values represent a potential set of default values, we strongly suggest users applying our approach explore the impact of these thresholds independently, as each dataset may have unique aspects that cause the filling algorithms to perform better with different thresholds.

**S.2 Despeckling algorithm thresholds**

The purpose of the despeckling algorithm is to remove highly unusual pixels when there are no similarly unusual pixels nearby. In practice, if users are very confident in the quality of the raw imagery (i.e., all non-gap pixel values are considered valid, regardless of how atypical they are) the despeckling algorithm is unnecessary. If users are concerned with the validity of pixels, the despeckling algorithm can be run with parameters that vary according to that uncertainty. The two key thresholds impacting the despeckling algorithm are (1) the standard deviation threshold that triggers the neighborhood analysis that tests nearby cells for similarly unusual values, and (2) a z-score threshold that is used to compare the average neighborhood z-score against the current pixel z-score. Lowering these two parameters increases the number of pixels that will be removed from the raw image and subsequently treated as gaps. The consequence of lowering these threshold values is a greater degree of smoothing in the final output images.

The appropriate value for the standard deviation parameter was determined as a by-product of an A1 sensitivity analysis, as the anniversary date images required by that model were not despeckled prior to use in this capacity. The results of this sensitivity analysis (Figure S1) indicate that the model accuracy for the combined model (i.e., the accuracy associated with all filled pixels, regardless of filling algorithm) stabilized with standard deviation threshold values of 2.58 and higher. In contrast to the standard deviation threshold, modifying the z-score threshold had essentially no impact on the overall gap-filling accuracy unless it was very low (e.g than 0.1) and thus removed many pixels, or so high (e.g., more than 0.6) that the despeckling algorithm removed very few cells. However, in preliminary phases of this research we found that a z-score threshold of +/-0.2 was sufficient for removing problematic pixels while still preserving the vast majority (> 99%) of non-gap cells in the raw imagery. While the overall impact of despeckling was minor (i.e., the overall accuracy changed by less than 1% when calculated on a continental scale), this processing step was effective for reducing the visually incongruous “hot” and “cold” spots that occasionally occurred on the resulting gap-filled imagery.


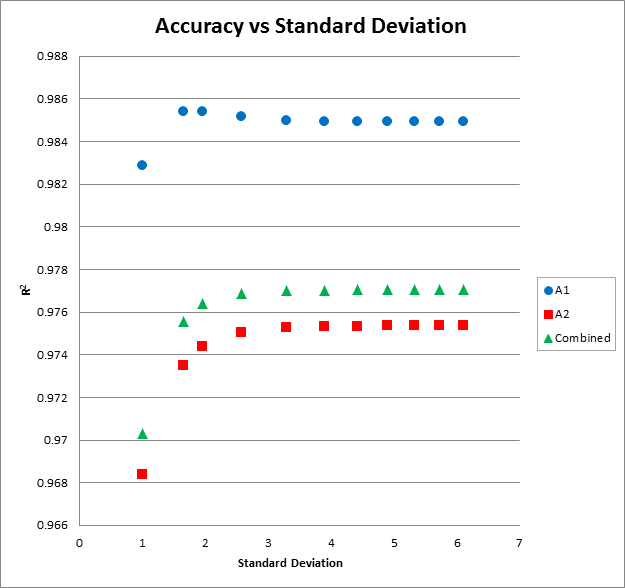


Figure S1: The relationship between the standard deviation threshold utilized in the despeckling and A1 algorithms for identifying, respectively, potentially erroneous pixels within the image being gap filled and the anniversary date images. Each plotted point represents the summarized metric associated with the filled values of all artificial gap pixels.

**S.3 A1 thresholds**

The A1 model is predicated on the concept of using neighboring pixels values and anniversary data information to model missing pixel values. The main considerations for this algorithm pertain to the search radius limit for finding usable neighbors, as increasing the radius size increases the run time of the model. The sensitivity analysis presented in Figure S2 shows the impact of the search radius size on modeled results when artificial gap stripes 25 km in width were introduced on the raw EVI image from day 129 of 2000. In general, the A1 model results behaved as expected, with decreasing accuracy as the radius increased, thus indicating that pixels farther from the edges of gaps were modeled with lower accuracy than pixels closer to the gap edge (a finding supported by the uncertainty analysis). Meanwhile, the A2 results were generally stable until the radius size reached approximately 9 km, after which the A2 model also displayed a distance-related decay in accuracy. Due to the cascading effect of pixels filled by A1 being used in A2, the accuracy of the combined output (i.e., overall results for all pixels regardless of model) show that the best overall result was achieved when radius was approximately 3.6 km in size. As such, a search radius of 3.6 km was used for gap filling the imagery time series processed for this research. This radius amounted to 40 possible neighboring cells, each with associated 12 associated anniversary dates, for a total of 1680 potential usable ratio pairs per gap pixel. An unexpected result of the sensitivity analysis is the finding that larger A1 search radii actually decrease the overall (combined) model accuracy to below the level achieved using only A2.


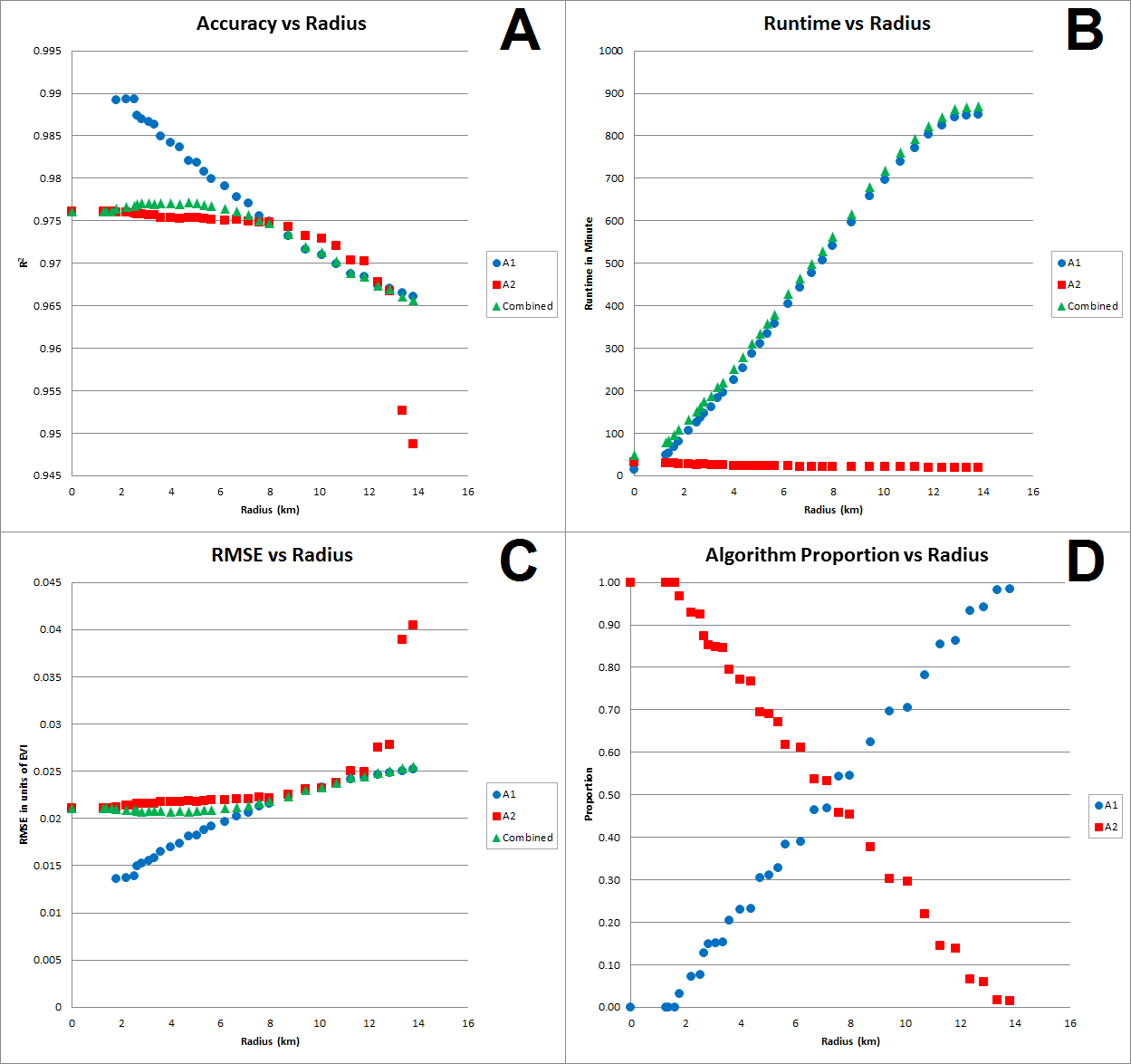


Figure S2: Results for the sensitivity analysis of the artificial gap pixels from the EVI image (day 129 of year 2000). Plots A and C show metrics of model accuracy, plot B shows the (per processing core, per image) run time associated with the gap filling models, and plot D shows the proportion of each filling algorithm utilized as a result of varying the A1 search radius size.
